# COVID-19 social distancing measures and informal urban settlements

**DOI:** 10.2471/BLT.20.265942

**Published:** 2021-04-01

**Authors:** Joyce Wamoyi, Meghna Ranganathan, Heidi Stöckl

**Affiliations:** a Department of Sexual and Reproductive Health, National Institute for Medical Research, Isamilo Road, 255 Mwanza, United Republic of Tanzania.; b Department of Global Health and Development, London School of Hygiene and Tropical Medicine, London, England.

To slow down the community transmission of severe acute respiratory syndrome coronavirus 2 (SARS-CoV-2), the virus that causes coronavirus disease 2019 (COVID-19), context-appropriate measures are vital. The first COVID-19 cases in Kenya, Uganda and the United Republic of Tanzania were reported in March 2020.^1^ All three countries have introduced measures to slow the spread of the virus, such as encouraging washing and/or sanitizing hands frequently, staying at home, practising physical distancing when outside the home and partial lockdowns. Most of these prevention measures replicate those from high-income countries and implementation in these three countries has not been without added challenges.^2^ The blanket transfer of prevention measures to low-income countries is problematic, especially in urban informal settlements in eastern Africa, due to major contextual differences.^3^^,^^4^ Here we draw on examples of these three East African countries to illustrate the challenges that individuals and families face in adopting measures such as physical distancing to slow down the spread of SARS-CoV-2.

## Social distancing

SARS-CoV-2 transmission mainly happens through the community. The current patterns of spread in Kenya and Uganda’s urban settings indicate that poverty is fuelling the spread of COVID-19 in the community, with overcrowded urban settlements, busy markets and shared social spaces.^5^^,^^6^ The factors that increase community transmission of SARS-CoV-2 are also the underlying factors that pose a challenge for the adoption of preventive policies. Most East Africans depend on public transport to go to work or attend markets. Similarly, markets are focal points of trade where opportunities to keep recommended distances are limited. Living spaces and household facilities in informal settlements are often crowded and shared by multiple family members, making social distancing within a family nearly impossible. Families also share key social and hygiene-related amenities, such as water sources and toilet facilities, that lack good ventilation and are not disinfected regularly.^5^^,^^6^ These shared resources also cause difficulties for citizens to efficiently practise physical distancing and hamper frequent handwashing, a simple and effective measure to slow down the spread of the pandemic.

## Economic toll

Although physical isolation or staying at home has been identified as a key measure for reducing the spread of SARS-CoV-2, the measure has exacted a heavy toll on people’s economic opportunities and survival options.^3^^,^^4^^,^^6^ Physical isolation measures threaten the economic existence of many individuals, who might therefore be less willing to make economic sacrifices and stay at home. Moreover, they may simply be unable to do that, as a lack of income means they cannot obtain basic food for their family, essential medications or other items needed for their day-to-day survival. While stockpiling food and other essential items to prepare for prolonged closures or restrictions have been frequently reported in high-income countries, this strategy is not an option for many people who survive on daily wages. In Kenya and Uganda, where the government provided relief packages of food and cash transfers, these and other social protection measures have still been inadequate for poor households to practise self-isolation.^2^^,^^6^ While physical distancing measures will reduce the spread of SARS-CoV-2, a major concern is that the cost of these measures on hunger-related morbidity and mortality in poor families in urban settings will be high. For most daily wage-earners, those in the informal sector and casual labourers, concerns about contracting SARS-CoV-2 come second to ensuring their livelihood and that of their family. Thus, in Uganda, for instance, the opportunity cost of lost economic opportunity far outweighs the risk and the fear of contracting the virus.^6^

## Challenges for parents and children

In Kenya, Uganda and the United Republic of Tanzania, which have weak social protection systems, tremendous financial and economic stress is placed on households, particularly on daily wage-earners. By losing out on financial opportunities, household wage-earners are unable to provide for the basic needs of their families, which may affect the overall social and physical well-being of family members, especially children, and increase intimate partner violence and child abuse. Financial stress and hunger are often main predictors of intimate partner violence.^7^ An inability to provide for essential family needs due to lockdown is disempowering to parents.^8^

Moreover, as many families struggle to fulfil their basic needs, adolescent girls and young women’s vulnerability to risky sexual behaviours might increase, such as engagement in transactional sex for food and other basic items to survive.^9^ School closures expose children in poor urban settings to abuse, hunger and malnutrition in situations where the main wage-earners have become unemployed due to the lockdown and physical distancing measures. Partial lockdowns in Kenya and Uganda have resulted in new household dynamics.^6^^,^^10^ While spending time together might initially be positive for couples, the increasing pressures on households could lead to violence. In families that already had an underlying history of violence, physical isolation could increase the control of an abusive partner, or of a parent over their children, and risk an escalation of violence.

## Recommendations

As SARS-CoV-2 continues to spread in Africa, measures to curb the transmission need to be tailored to the contextual realities of the individual countries and specific populations. Structural and practical limitations of physical distancing in poor urban settings in East Africa exist, as prevailing social norms encourage family togetherness and sharing of important services. Adopting the concept of physical distancing implies the acceptance of new social norms by many families.

Therefore, since community spread is the main mode of transmission, implementing measures that consider the realities of people’s lives in informal urban settlements is essential. Immediate actions for poor informal urban settlements or in crowded markets are feasible, such as deploying handwashing stations, regular cleaning of surfaces, mandatory wearing of masks in indoor spaces and limited physical contact when socializing outside the family setting. Longer-term actions involve addressing poverty, alongside physical distancing measures, for these to be effectively implemented. As of 18 September 2020, a total of 212 countries have planned, introduced or adapted social protection measures in response to COVID-19.^10^ Programmes such as emergency cash transfers to households, including to women, can immediately provide support to meet basic needs such as food and health care. Simultaneously, these programmes should aim for long-term goals through improved income – hence better living conditions – including decent accommodation. Furthermore, in-kind voucher schemes and school feeding programmes should be expanded to protect vulnerable children. Seeing some of these responses already being implemented in East Africa is encouraging; they should be expanded further. Imposing strategies that have been used in high-income countries on low-income countries without much consideration for contextual differences is counterproductive in slowing the spread of the virus. Governments in East Africa should adopt measures that are in line with the contextual realities of the crowded urban settings of their countries.
